# Pharmacological iron-chelation as an assisted nutritional immunity strategy against *Piscirickettsia salmonis* infection

**DOI:** 10.1186/s13567-020-00845-2

**Published:** 2020-10-28

**Authors:** Mario Caruffo, Dinka Mandakovic, Madelaine Mejías, Ignacio Chávez-Báez, Pablo Salgado, Daniela Ortiz, Liliana Montt, Javiera Pérez-Valenzuela, Francisca Vera-Tamargo, José Manuel Yánez, Jurij Wacyk, Rodrigo Pulgar

**Affiliations:** 1 Laboratorio de Genómica y Genética de Interacciones Biológicas (LG2IB), Instituto de Nutrición y Tecnología de los Alimento, Universidad de Chile, Av. El Líbano 5524, Macul, 7830490 Santiago, Chile; 2 Escuela de Biotecnología, Facultad de Ciencias, Universidad Santo Tomás, Santiago, Chile; 3 Center for Research and Innovation in Aquaculture (CRIA), Universidad de Chile, Santiago, Chile; 4 Laboratorio de Nutrición Animal (LABNA). Facultad de Ciencias Agronómicas, Producción Animal, Universidad de Chile, Santa Rosa, 11315 La Pintana, Chile; 5 Facultad de Ciencias Veterinarias y Pecuarias, Universidad de Chile, Santa Rosa, 11735 La Pintana, Chile; 6 Laboratorio Inmunología en Peces, Facultad de Ciencia de la Vida, Universidad Andrés Bello, República 239, Santiago, Chile; 7 GEMA Center for Genomics, Ecology and Environment, Universidad Mayor, Camino La Pirámide 5750, Huechuraba, Santiago, Chile; 8 Laboratory for Research in Functional Nutrition, Instituto de Nutrición y Tecnología de los Alimentos, Universidad de Chile, Av. El Líbano 5524, Macul, 7830490 Santiago, Chile; 9 Scimetrica Lab, Santiago, Chile

**Keywords:** *Piscirickettsia salmonis*, host-directed therapy (HDT), iron-chelator, deferiprone (DFP), infection

## Abstract

Salmonid Rickettsial Septicaemia (SRS), caused by *Piscirickettsia salmonis*, is a severe bacterial disease in the Chilean salmon farming industry. Vaccines and antibiotics are the current strategies to fight SRS; however, the high frequency of new epizootic events confirms the need to develop new strategies to combat this disease. An innovative opportunity is perturbing the host pathways used by the microorganisms to replicate inside host cells through host-directed antimicrobial drugs (HDAD). Iron is a critical nutrient for *P. salmonis* infection; hence, the use of iron-chelators becomes an excellent alternative to be used as HDAD. The aim of this work was to use the iron chelator Deferiprone (DFP) as HDAD to treat SRS. Here, we describe the protective effect of the iron chelator DFP over *P. salmonis* infections at non-antibiotic concentrations, in bacterial challenges both in vitro and in vivo. At the cellular level, our results indicate that DFP reduced the intracellular iron content by 33.1% and *P. salmonis* relative load during bacterial infections by 78%. These findings were recapitulated in fish, where DFP reduced the mortality of rainbow trout challenged with *P. salmonis* in 34.9% compared to the non-treated group. This is the first report of the protective capacity of an iron chelator against infection in fish, becoming a potential effective host-directed therapy for SRS and other animals against ferrophilic pathogens.

## Introduction

Infectious diseases are responsible for considerable economic losses in salmon farming. Salmonid Rickettsial Septicaemia (SRS) is a severe disease that has generated losses up to USD 450 million per year in the Chilean salmon farming industry [1, 2]. *Piscirickettsia salmonis*, the etiological agent of SRS, is a Gram-negative intracellular facultative bacterium [3, 4] that can replicate and propagate in several fish cells, including salmonid macrophages [5].

Vaccines and antibiotics are the current strategies for prevention and treatment to fight SRS. It has been reported that *P. salmonis* is the main bacterial pathogen for which a high amount of antibiotics is being used in the Chilean salmon farming industry [6]. Furthermore, this strategy of pathogen-directed antimicrobial drug (PDAD) targeting is associated with increased microbial drug resistance and therefore, a resurgence of infectious diseases [7]. In the case of SRS, strains of *P. salmonis* resistant to quinolones have already been reported [8]. In this regard, the limited effectiveness of current management, prevention and treatment strategies, confirms the need to develop new strategies to combat this disease.

An innovative therapeutic approach to treat infectious diseases produced by intracellular pathogens is to perturb host pathways used by the microorganisms to enter, replicate and/or survive inside host cells through host-directed therapies (HDT). The use of host-directed antimicrobial drugs (HDAD) facilitates overcoming antimicrobial resistance [9] and permits the testing of drugs that were designed to treat conditions other than infections (drug repositioning) [10, 11]. The difficulty of this approach is identifying host metabolic pathways or biological processes relevant for the development of infection; however, key knowledge of host–pathogen interactions between salmonid fish and *P. salmonis* that denote potential targets for HDT is already available.

Iron is an essential nutrient for *P. salmonis* since its growth in culture depends on its supplementation [12–14]. In fact, several pathways for iron acquisition have been described for *P. salmonis* [15, 16]. Furthermore, in a previous study, we reported that iron deprivation/withholding is an innate immunity mechanism through which resistant families of Atlantic salmon, but not susceptible families, can limit the iron available for *P. salmonis*, inhibiting its proliferation and highlighting the relevance of iron for bacterial pathogenesis [15]. Since not all farmed fish are genetically resistant, this iron deprivation could be induced through a pharmacological strategy based on the use of host-directed iron chelators.

In humans, iron chelation therapy is used to reduce genetically or acquired iron overload observed in various organs such as the liver, brain and heart [17, 18]. The FDA-approved iron chelators differ in their routes of administration, stoichiometries, doses and routes of excretion. For instance, Deferoxamine (DFO) is a hexadentate chelator, which should be parenterally applied since it is not well absorbed from the gastrointestinal tract [19, 20], while Deferasirox (DFX) and Deferiprone (DFP) are oral tri- and bidentate iron chelators, respectively [21]. Furthermore, cost-effectivity differences in iron chelation therapies have been reported, indicating that DFP was the most profitable [22]. Despite these differences, the three iron chelators were tested as therapeutic agents against mammalian pathogens, showing divergent results. Deferiprone appears to have the highest therapeutic and protective capacities for antimicrobial activity against ferrophilic bacteria, fungi, protozoa and viral infections compared with DFO and DFX [23–27], emphasizing the relevance of selecting an adequate iron chelator to the specific characteristics of each infection. In sum, DFP is an oral FDA-approved iron chelator that has been shown to be the most cost-effective against mammalian infections, making it a suitable candidate for massive use in productive industries.

In the present study, we investigated which non-antibiotic concentrations of DFP against in vitro and in vivo* P. salmonis* infections demonstrate its capacity as HDAD against SRS. This investigation gives the first insights into a potential effective host-directed therapy for salmonids and other fish against ferrophilic pathogens, such as *P. salmonis*.

## Material and methods

### Cells and bacterial culture conditions

The SHK-1 cell line (ECACC 97111106) was obtained from the European Collection of Authenticated Cell Cultures (ECACC). Cells were cultivated at 18 °C in Leibovitz L-15 Medium (Gibco, USA) supplemented with 5% of inactivated fetal bovine serum (FBS Gibco) and 40 μM of 2-mercaptoethanol in T-25 flasks (Corning). Cell viability was quantified using the trypan blue exclusion assay [28]. *Piscirickettsia salmonis* LF-89 (ATTC VR-1361) used in this study was obtained from the American Type Culture Collection (ATCC) and was cultivated at 18 °C in solid and/or liquid SRS-broth media [12] with constant stirring of 180 rpm. Each subculture was confirmed as *P. salmonis* by Gram staining and RFLP assay [29]. After 4 days, cultures were diluted to an optical density (OD_620_) of 0.05 in 5 mL of liquid media and incubated at 18 °C. Absorbance was measured in an Infinite® 200 PRO NanoQuant (Tecan®) equipment. For treatment with DFP, bacteria and cells were independently cultured in the conditions indicated above and media was supplemented with DFP concentrations ranging from 0 to 500 µM.

### In vitro infections

SHK-1 cells were seeded on coverslips and cultured with Leibovitz L-15 medium supplemented with 5% of FBS (Gibco) without antibiotics in 24-well plates (Corning). Twenty-four hours later, cells approximately 80% confluent, were supplemented with 25 µM of DFP (and controls with no DFP supplementation) and inoculated with stationary phase bacteria at a multiplicity of infection (MOI) of 100 (1 cell: 100 bacteria) (controls not inoculated with bacteria were also cultured). After 24 h, cells were washed twice with cold PBS and then incubated for 60 min with L-15 medium plus gentamicin (100 µg/mL) to eliminate extracellular bacteria [30]. After incubation, cells were washed with PBS and incubated in L-15 medium supplemented with or without DFP as indicated above. To evaluate the cytopathic effect (CPE) caused by *P. salmonis*, SHK-1 cells were stained with hematoxylin–eosin (H&E) [31] and observed under an optical microscope (Inverted Phase Contrast Microscope Motic AE31) to follow the progression of the infection using image analysis (Moticam BTU10). Ten days post-inoculation (10 dpi) with *P. salmonis*, images of cells of ten fields randomly obtained for each condition, were used to count vacuoles and measure their sizes. Cell viability was quantified using the trypan blue exclusion assay (Gibco).

### Iron content quantification

To measure intracellular iron, SHK-1 cells were seeded at a density of 1 × 10^6^ and grown for 24 h before exposition to 0 or 25 µM DFP for 10 days. Afterwards, cells were washed three times with 1 mL of PBS and 1 mM EDTA at 4 °C and harvested by trypsinization. For tissue iron quantification, 50 to 100 mg of five samples of head kidney obtained at the end of the challenge (S5, sampling point 5) from non-treated and treated with DFP fish, were dried by evaporation (SpeedVac, Thermos) and processed as described previously [15]. Aliquots of SHK-1 cells and dried head kidney tissues were digested with nitric acid 65% (Merck) for 14 h at 80 °C and intracellular iron levels were measured using an atomic absorption spectrophotometer with graphite furnace (SIMAA 6100, Perkin Elmer). An aliquot of cells was kept for protein determination by Bradford assay. Total intracellular iron concentration in SHK-1 was expressed as nmoles of iron per mg of protein, while head kidney iron concentration was expressed as µg of iron per mg of dried weight.

### *Piscirickettsia salmonis* quantification in infected SHK-1 cells

To isolate RNA from intracellular growing *P. salmonis*, monolayers of infected SHK-1 cells were rinsed twice with cold PBS and treated with 0.25% trypsin–EDTA solution. Cells were resuspended in 1 mL of TRI Reagent (Invitrogen) according to the manufacturer’s instructions, and then incubated for 30 min at 37 °C with 20 units of RQ1 RNase-Free DNase (Promega) to remove residual genomic DNA. RNA was purified using RNeasy mini kit (Qiagen) according to the manufacturer’s instructions. The quantity of the total RNA was determined using a Qubit Fluorometric Quantitation System (Life Technologies) and the purity (absorbance 260/280 nm) using a NanoQuant Spectrophotometer (Tecan Technologies). Two μg of total RNA were used as the template for reverse transcription reactions to synthesize cDNA using High-Capacity RNA to cDNA Kit (Thermo Fisher Scientific), according to standard procedures. cDNA were diluted to 100 ng and used as templates for qPCR, reactions that were carried out on a real-time PCR System (Roche) using Terra qPCR Direct TB Green Premix (Takara). Briefly, PCR conditions were 95 °C for 5 min followed by 94 °C for 15 s, 60 °C for 15 s and 72 °C for 20 s for a total of 35 cycles using primers for *P. salmonis* 16S RNA gene previously reported [29]. Previously reported [15] primers for elongation factor 1 alpha gene (EF1A) of Atlantic salmon and rainbow trout were selected as the normalizer gene. At least five replicates were performed for each *P. salmonis* quantification.

### Fish experiments

#### Fish acclimatization and diet preparation

Disease-free *Oncorhynchus mykiss* of 55 g average weight were obtained from a local aquaculture facility and maintained at the Quillaipe experimental center (Puerto Montt, Chile). Before trials, fish were acclimated to a controlled environment in 1 m^3^ tanks at a density of 18 kg/m^3^ in fresh water with an exchange rate of 0.8–1 m^3^ / hour and water salinity was gradually increased to ~ 32 parts per thousand (ppt previous to the challenge). Water condition during acclimatization was the following: 10.7 ± 0.95 °C and oxygen saturation of 84.8–112%. Thirty-five days after acclimation, selected groups began to receive DFP-supplemented feed in two concentrations 50 or 100 mg/Kg (DFP 50 BC and DFP 100 BC, respectively) per day, while the rest of the tanks received the same diet without DFP. Ten days after the feeding with the DFP-diet, all fish were placed in 180 L tanks where the experiments and challenges were conducted. All fish were fed at 1% body weight per day with an extruded trout diet either with or without DFP as appropriate.

Diets were prepared as indicated in Additional file 1 using as a base the commercial diet manufactured by Salmones Antártica (Puerto Montt, Chile). Experimental diets were prepared by supplementing with DFP at three different concentrations, 0, 5 and 10 g DFP per Kg of diet (DFP −, DFP 50 and DFP 100, respectively). DFP was dissolved and homogenized in fish oil and then incorporated into dry pellets using a laboratory vacuum coater. Diets were isoenergetic and isonitrogenous and met National Research Council nutritional requirements for rainbow trout [32]. Proximal composition analyses of the diet were performed at the Instituto de Nutrición y Tecnología de los Alimentos (INTA, Universidad de Chile) according to the following procedures: dry matter was obtained after 24 h in an oven at 105 °C; ash by combustion at 450 °C for 16 h, protein (N*6.25) by the Kjeldahl method; fat by the Soxhlet method; and gross energy by calorific factor (4, 9 and 4 for proteins, lipids and carbohydrates, respectively).

#### Experimental design

Before the challenge experiment, the median lethal dose (LD_50_) of *P. salmonis* LF-89 was determined. *Piscirickettsia salmonis* inoculum was provided by ADL Diagnostic Chile Ltda. Five dilutions were assessed from a stock concentration of 1 × 10^5^ tissue culture infective dose 50% per mL (TCID_50_/mL, determined through the Spearman-Kärber method), from which five dilutions were made by a factor of 10. These dilutions, plus a control made up of L15 media (Leibovitz, Invitrogen), were administered by intraperitoneal (IP) injection in a final volume of 200 μL. Fish were distributed in 180 L tanks (40 fish/tank). Water conditions during LD_50_ were the following: 15.1 ± 0.11 °C and oxygen saturation of 88.7–116.2%. Mortality was registered daily until 26 days post-challenge.

Seventy fish per group were stocked in 180 L tanks at a density of 38 kg/m^3^ in seawater. Water conditions during the challenge were the following: 14.5 ± 0.25 °C and oxygen saturation of 88.3–115.3%. Experimental design comprised three feeding groups: 0 (control), 50 and 100 mg DFP/Kg fish per day and two different delivery strategies: before and after challenge (or sampling 0, S0) (Additional file 2). These groups were challenged with 200 μL of the *P. salmonis* inoculum with the highest mortality in LD_50_ (1:10 of 1 × 10^5^ TCID_50_/mL) or injected with 200 μL of sterile L15 culture media representing the control condition (non-infected fish), as previously reported [15]. Experimental diets, delivery strategies and challenges were developed in independent tanks with two replicates per treatment and mortality was recorded daily until day 30 post-challenge.

#### Sampling

The sampling strategy was designed using the LD_50_ (Additional file 3) with the aim of taking samples at different stages of the curve of mortality (before the challenge, before deaths begin, after deaths begin, during the active death phase and at the end of the challenge). Defining six sampling points at different stages of the disease (S0, S1, S2, S3, S4, S5; corresponding to days after challenge 0, 4, 8, 16, 23 and 30, respectively). Blood and kidney tissues were sampled from five fish euthanized by an overdose of benzocaine (Sigma) (20% w/v; 50 mg/L) per group as previously described sampling points to evaluate bacterial load, plasma iron levels and gene expression. Kidney tissues were stored at -20 °C in RNA Later (Ambion, USA). Blood samples were drawn from the caudal vein using 5 mL syringes with 21 G needles and collected in heparinized tubes (4 mL, BD Vacutainer®, NJ USA. 68 USP). Plasma separation was performed by centrifugation at 3500 rpm for 10 min. Samples were stored at -20 °C or 4 °C until use. Sampling and mortality monitoring were performed in parallel experimental groups to avoid the influence of stress on mortality due to handling.

### Bactericidal effect and iron content in plasma

To determine the effect of plasma on bacterial growth, *P. salmonis* was cultured as indicated above in liquid media SRS-broth replacing fetal bovine serum with plasma sampled in S5 from non-treated and DFP treated fish. The carrying capacity (k) of *P. salmonis* was quantified at the stationary phase of bacterial growth at 5 days post-inoculation. To determine the plasma iron content, 300 μL of plasma sampled in S0 to S5 were assayed in an automatic biochemistry analyzer CM250 (Wiener Lab) using the FeR-color kit (ID 861272522 Wiener Lab) according to the manufacturer’s instructions.

### Gene expression of iron metabolism markers

Reactions were carried out on a real-time PCR System (Roche) using the Terra qPCR Direct TB Green Premix kit (Takara). Total RNA was extracted from 50 to 100 mg of head kidney tissue using TRIZOL Reagent (Invitrogen), and incubated for 30 min at 37 °C with 20 units of RQ1 RNase-Free DNase (Promega) to remove residual genomic DNA. Then, RNA was purified using RNeasy mini kit (Qiagen) and RNA concentration was determined as described above. Two μg of total RNA was used as the template for reverse transcription reactions to synthesize single strand cDNA using High-Capacity RNA to cDNA Kit (Thermo Fisher Scientific), according to standard procedures. cDNA were diluted to 100 ng and used as the template for qPCR, with primers designed against three markers of cellular iron status: transferrin receptor (*TfR*), ferritin (*ferH*) and iron-regulated transporter ferroportin 1 (*ireg1*). Briefly, PCR conditions were 95 °C for 5 min followed by 94 °C for 15 s, 60 °C for 15 s and 72 °C for 20 s for a total of 35 cycles. Primers for elongation factor 1 alpha gene (EF1A) was used as the normalizer gene as previously reported [15]. To determine relative expression levels of genes, the method described by Pfaffl [33] and adapted by Talke [34] was used. The primers used are listed in Additional file 4.

### Data analysis

Statistical analysis was performed using the software GraphPad Prism 8 (Graphpad Software, Inc). Differences in bacterial growth, cell viability and relative *P. salmonis* load using different DFP concentrations and/or sampling points were analyzed using two-way analysis of variance (ANOVA) followed by Bonferroni comparison test. Differences in bacterial growth, CPE cells/total cells and *P. salmonis*-containing vacuoles (PCV) area obtained at a specific time point and at a defined concentration of DFP (25 μM), were analyzed using one-way ANOVA and Tukey multiple comparisons test. Data from intracellular iron, activity of plasma on bacterial growth, kinetic of plasma iron levels and gene expression were analyzed using unpaired t-tests.

Survival curves were analyzed using Kaplan–Meier and group differences were analyzed using Log-rank test. To assess the effectiveness of formulations, the relative percent survival (RPS), absolute risk reduction (ARR), and number of animals necessary to treat (NNT) were calculated (all formulas used are described in Additional file 5). p-value < 0.05 was considered significant.

## Results

### Deferiprone decreases intracellular iron content in SHK-1 at non-bactericidal nor cytotoxic concentrations.

**Figure 1:**
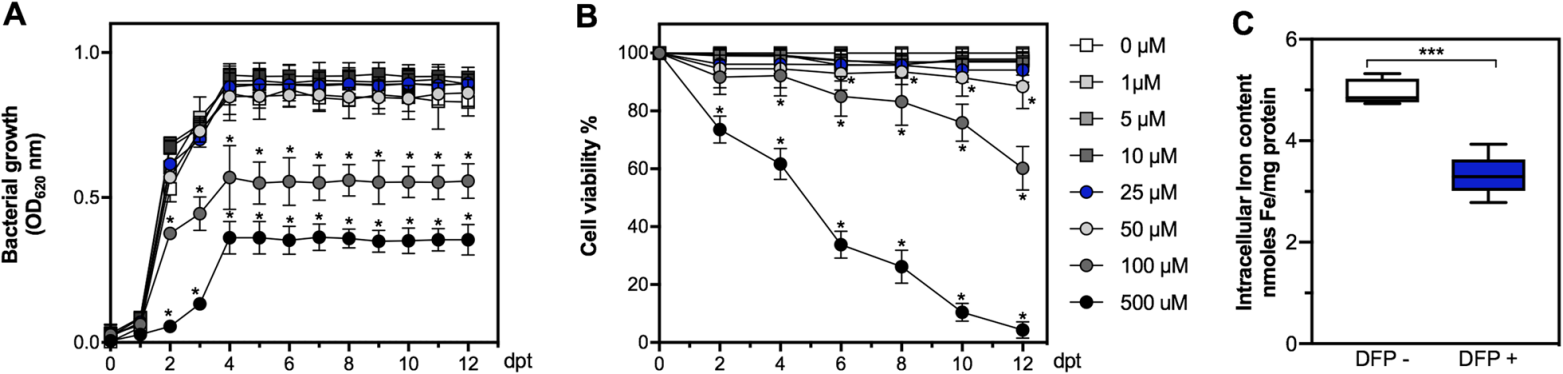
**Effect of Deferiprone on** ***Piscirickettsia salmonis*** **growth and SHK-1 cell viability**. **A** Bacterial growth of *P. salmonis* at different concentrations of DFP supplemented in SRS broth. **B** SHK-1 viability at different concentrations of DFP supplemented in SRS broth. **C** Determination of intracellular Fe (nmoles Fe/mg protein). Experiments of bacterial growth and cell viability were performed until 12 days post-treatment (dpt), and each circle represents the mean ± SD of nine observations measured in at least three independent experiments. Experiment of iron content was performed 10 days post-treatment (dpt), data show box and whisker plot and solid horizontal lines indicate the median and the 25th 75th interquartile range level of at least five replicates. In **A** and **B** Two-way ANOVA and subsequent Bonferroni comparison test relative to control (0 µM DFP) were performed. In **C** unpaired t-test was performed between groups. Asterisks show significant differences (p-value < 0.05).

In order to determine the maximum concentrations of DFP that do not generate an antiproliferative or cytotoxic effect on *P. salmonis* and SHK-1 cells, we cultivated bacteria and cells independently with supplementation of DFP at different concentrations (0–500 μM) for 12 days. Deferiprone did not generate a bactericidal effect when *P. salmonis* was grown at concentrations ranging from 0 to 50 μM. Nonetheless, we observed antiproliferative effects at concentrations greater than 100 μM (p-value < 0.05) from the second day of cultivation (Figure 1A). Similarly, we recorded no effects on cell viability of SHK-1 cells at concentrations lower than 25 μM, while concentrations greater than 50 μM showed reduced cell viability in a dose- and time-dependent manner (p-value < 0.05; Figure 1B). Therefore, the maximum concentration evaluated that did not cause a reduction in *P. salmonis* proliferation and SHK-1 cell viability was 25 μM of DFP; hence this concentration was selected to perform the following in vitro experiments. Also, to determine the effect of DFP on the iron content in SHK-1 cells, we measured the intracellular iron content by Atomic Absorption Spectroscopy (AAS). The results indicate that 25 μM of DFP reduced iron content by 33.1% (from 4.96 to 3.32 nmoles Fe/mg protein) after 10 days of exposure to the chelator (Figure 1C).

### Deferiprone reduces *Piscirickettsia salmonis* infection in SHK-1 cells.

**Figure 2:**
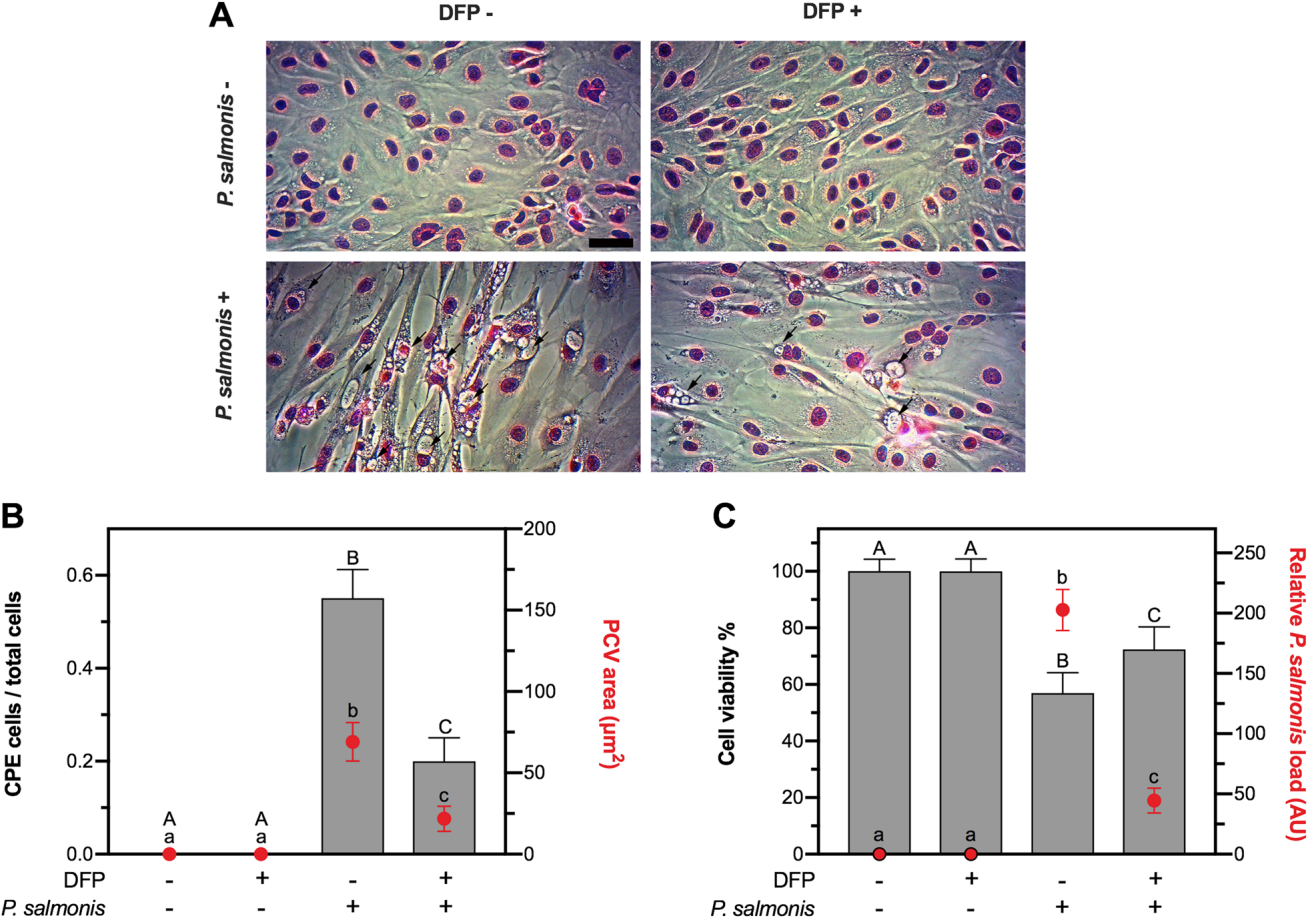
**Effect of Deferiprone in SHK-1 cell viability infected with** ***Piscirickettsia salmonis***. **A** Representative microphotograph of SHK-1 monolayer exposed (DFP +) or not (DFP −) to DFP (25 μM) in panoptic staining. Upper panels show uninfected cells and lower panels *P. salmonis* infected cells after 10 days of infection and DFP treatment, Bar = 10 μm. **B** The ratio of cytopathic (CPE) on total cells (left axis) in grey bars and area of *P. salmonis* containing vacuoles (PCV) represented by red circles in SHK-1 DFP treated/untreated and infected/uninfected cells. **C** Cell viability percentage (left axis) in grey bars and relative *P. salmonis* load (arbitrary units, AU) (right axis) represented by red circles, in SHK-1 DFP treated/untreated and infected/uninfected cells. For **A** and **C**, data represent mean ± SD of 10 observations measured in at least three independent experiments. One-way ANOVA and Tukey multiple comparisons between all treatments were performed; different letters represent significant differences (p-value < 0.05; capital letters for left axis and lowercase letters for right axis comparison).

To investigate whether DFP exerts a protective effect on infection, we performed an in vitro infection assay of SHK-1 cells challenged with *P. salmonis* during 10 days with (25 μM) and without DFP. We characterized the infection according to the cytopathic effect (CPE) displayed by the infected cells, a phenomenon that is described by the presence of replicative *P. salmonis*-containing vacuoles (PCV) in SHK-1. The results display an evident decrease of PCV in cells inoculated with *P. salmonis* and treated with DFP compared to those not treated with the iron chelator (Figure 2A). The quantification of infected cells treated with DFP shows a significant reduction of both the number of cells exhibiting CPE from the total (63.7% reduction) and the size of PCV (68.5% reduction) (Figure 2B). Moreover, SHK-1 infected cells reduced their viability up to 57% with respect to non-infected control cells in the absence of DFP; however, when the medium was supplemented with DFP, infected cells incremented their viability up to 72.4% (Figure 2C). Thus, a significant increase in cell viability was observed in response to DFP on infected cells (p-value < 0.05).

Interestingly, the increased cell viability observed in the DFP-treated infected group correlated with a lower number of bacteria (44.5 arbitrary units, AU) compared with the non-treated infected group (202.7 arbitrary units, AU) (Figure 2C). Taken together, these results indicate that DFP, at a non-antibiotic and non-cytotoxic concentration, reduces the cytopathic effect, the infection progression and the mortality of SHK-1 cells infected with *P. salmonis*. Furthermore, the results suggest that the iron chelator exerts a protective effect by reducing the availability of intracellular iron to *P. salmonis*, affecting the bacterial proliferative capacity.

### Deferiprone reduces mortality of *Oncorhynchus mykiss *challenged with *Piscirickettsia salmonis*.

To test whether the results of DFP protection under in vitro conditions could be recapitulated in vivo, we fed rainbow trout (*Oncorhynchus mykiss*) with standard growth diets supplemented with DFP in three different concentrations (0, 50 and 100 mg DFP per Kg of fish) and used two different delivery strategies (before (BC) or after (AC) challenge with *P. salmonis* or sampling 0, S0; Additional file 2). Our aim was to expose the bacterium to different scenarios through two strategies, (1) a preventive approach, where DFP began to be delivered 10 days before the challenge (BC), and (2) a therapeutic approach, where DFP commenced to be delivered one day after the challenge (AC). In both delivery strategies, DFP was distributed daily in the diet until the end of the challenge trial that lasted 30 days, and five sampling points (S0–S5) were selected to characterize the infection (Additional file 2). A group of fish subjected to the same conditions but not challenged with the bacterium (control group) was also evaluated.

**Figure 3:**
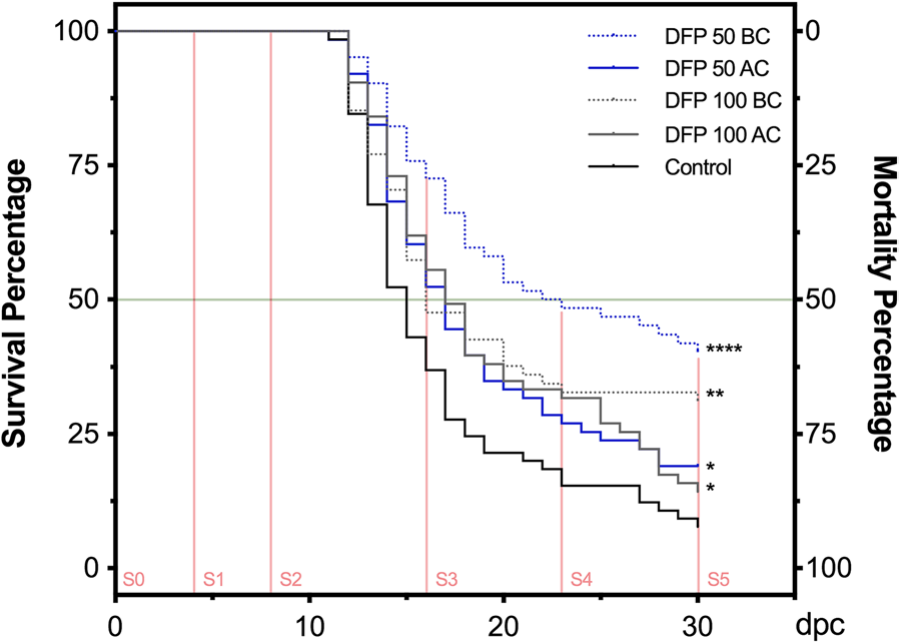
**Effect of Deferiprone on survival of** ***Oncorhynchus mykiss*** **challenged with** ***Piscirickettsia salmonis. ***The data show the survival percentage of fish subjected to two concentrations of DFP in feed (50 and 100 mg/Kg per day; DFP 50 blue lines and DFP 100 gray lines, respectively) and two delivery methods: 10 days before challenge (BC, dotted line) and immediately after challenge (AC, continuous line). Survival was monitored on a daily basis for 30 days. Kaplan–Meier and subsequent survival curve comparison by Log-rank test was performed. Asterisks show statistically significant differences between DFP treated and untreated (control black line) fish and denote: *p-value < 0.05, **p < 0.01, ****p < 0.0001. The horizontal light green line denotes the 50% of survival percentage and the vertical light red lines represent the sampling points (S0–S5) at different days post-challenge (dpc). The experiments were performed in duplicate tanks and representative data is shown.

Notably, the results show that at 30 days post-challenge with *P. salmonis* (30 dpc), in all fish fed with DFP, there was a significant reduction of mortality compared with the non-treated control group, which had a mortality of 91.6% (Figure 3). The groups that received the treatment before the challenge (BC) had the lowest percentages of mortality of 59.6 and 68.9% for DFP 50 BC and DFP 100 BC, respectively, while the groups that received DFP after the challenge (AC) had an 81 and 85.7% mortality for DFP 50 AC and DFP 100 AC, respectively. These different levels of protection were evident early in the trial (14 dpc) and remained, or were even accentuated, until termination. Moreover, to complement these results and further characterize the efficacy of DFP on SRS protection, the relative percentage survival (RPS) and the absolute risk reduction (ARR) were calculated at the end of the trial. These indicators estimate the risk reduction of death by *P. salmonis* in the different treatments (in relative terms for RPS and in absolute terms for ARR). We also calculated the number of animals necessary to treat (NNT), an indicator that indicates the number of animals that must receive the treatment (DFP) in order for one animal to survive the evaluated time interval (30 days) (Additional file 6). Treated groups that received DFP ten days before the challenge at a concentration of 50 mg/Kg (DFP 50 BC) presented an RPS of 34.93%, ARR of 32.63% and NNT = 4, while and at a concentration of 100 mg/Kg (DFP 100 BC), fish presented an RPS of 24.78%, ARR of 23.46% and NNT = 5. Groups that received the iron chelator after the challenge (DFP 50 and 100 AC) had an RPS less than 11.6% and ARR lower than 11.4% with confidence intervals with negative lower limits. These results indicate that the time at which DFP is administered is more relevant than its concentration, since fish that received the treatment 10 days before the challenge had higher survival rates.

The results indicate that DFP protects fish from mortality associated with SRS, with greater efficiency in fish fed with the lowest concentration of the iron chelator evaluated and delivered before the challenge with *P. salmonis*. To expand in this aspect, we registered the food consumed by non-challenged fish under the four feeding conditions and the control diet (Additional file 7) and measured its effect on weight gain (Additional file 7) and survival (Additional file 8). As shown in Additional file 7, the diet consumed was significantly lower in the groups that received diets supplemented with DFP with respect to the control group, in a dose and time-dependent manner. Remarkably, this reduction in the diet feeding, which averaged 25.7%, resulted in a decrease of only 8.2% in average body weight of fish when compared with the control group. Moreover, fish fed with the diet with the lowest concentration of DFP and administered for the shortest time (DFP 50 AC), showed no significant difference in average body weight at the end of the experiment when compared with the control group, even though their feed consumption was 20% lower (Additional file 7). Additionally, no mortalities were observed in fish fed with diets supplemented with 50 mg DFP (DFP 50 diet), independent of the delivery strategy, while there was 5 and 10% mortalities in fish fed with diets supplemented with 100 mg DFP (DFP 100 diet), independent of the delivery strategy (Additional file 8). Altogether, these results emphasize the fine-tuning regulation of iron metabolism in salmonids and highlight the possibility of optimizing the design of diets supplemented with DFP to protect fish from SRS.

### Deferiprone decreases *Piscirickettsia salmonis* burden in fish kidney at a non-antibiotic concentration

**Figure 4:**
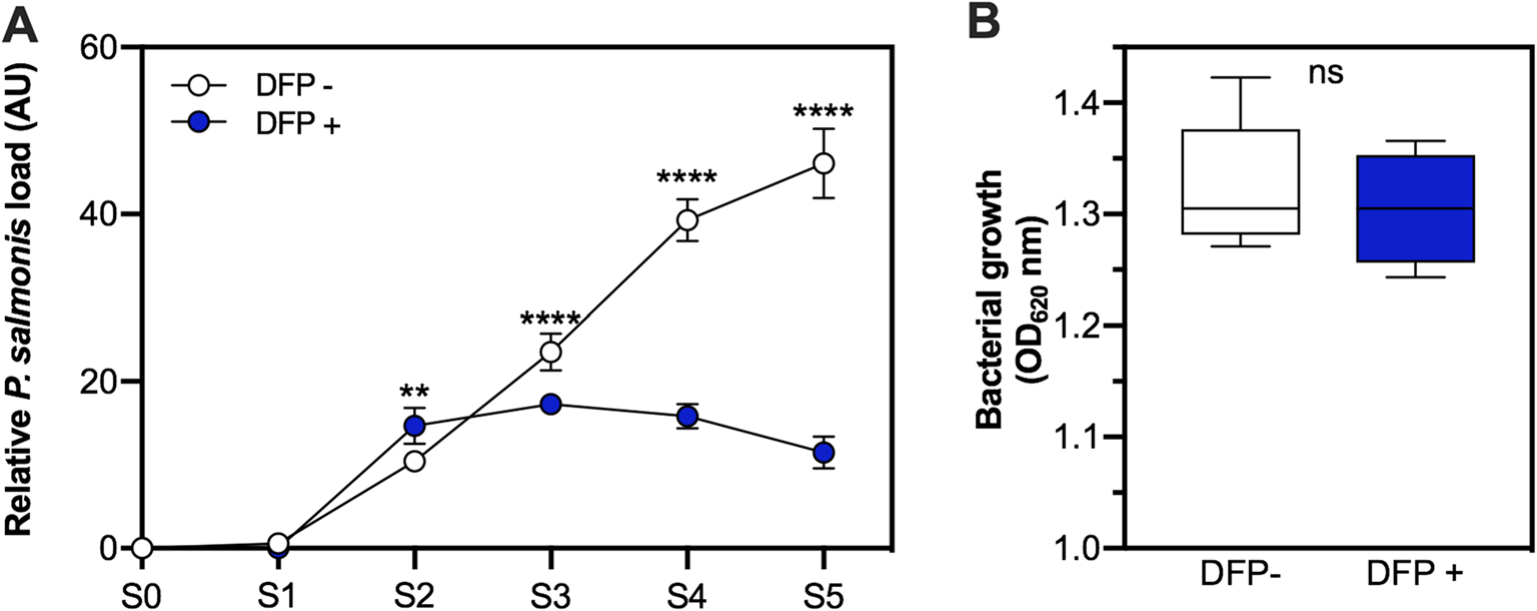
**Effect of Deferiprone on the bacterial burden in challenged fish anterior kidney and plasma from treated fish on bacterial growth**. **A** Relative *P. salmonis* load (arbitrary units, AU) in the anterior kidney from untreated (DFP −, white circles) or treated (DFP + , blue circles) fish during the course of the experiment at different S0 to S5 sampling points (0, 4, 8, 16, 23, 30 days post-challenge (dpc), respectively). Each point represents the mean ± SD of 5 fish measured in at least two independent experiments. Two-way ANOVA and subsequent Bonferroni comparison test relative to control (DFP −) was performed. Asterisks show statistically significant differences denote: **p-value < 0.01, ****p-value < 0.0001. **B** Bacterial growth of *P. salmonis* in SRS broth replacing fetal bovine serum with plasma from unchallenged and untreated (DFP −, white box) or treated (DFP + , blue box) fish and quantified at the stationary phase of bacterial growth (5 days post-inoculation). Data show box and whisker plot and solid horizontal lines indicate the median and the 25th 75th interquartile range level of at least five independent experiments. Unpaired t-test was performed between groups; ns = statistically non-significant.

To characterize the possible underlying mechanisms of DFP protection, DFP 50 BC group (the group with the highest survival percentage) was further analyzed. For this purpose, fish anterior kidneys from DFP 50 BC and control groups were sampled during the course of the trial (S0–S5) to evaluate the effect of the iron chelator on the bacterial burden. As shown in Figure 4A, and similar to what was observed for SHK-1 cells, the anterior kidney of fish fed with DFP revealed a lower bacterial load than fish fed without DFP (control). The load of viable bacteria was detected at 8 dpc (S2) in this tissue, where initially the relative concentration of the pathogen was higher in the DFP treated over the control group. However, from S3 until the end of the challenge (S5), the bacterial load was significantly lower in the DFP treated group than in the control. To assess if the chelator could exert its effect through bactericidal activity, we replaced fetal bovine serum of the broth culture of *P. salmonis* with plasma from unchallenged fish fed with or without DFP obtained at the end of the assay (S5). The carrying capacity (*k*) of *P. salmonis* in the stationary phase of its growth curve (5 days post-inoculation) shows no difference between the DFP treated and the control groups (Figure 4B). These results suggest that the inhibition of bacterial proliferation in fish tissues is due to the iron chelator function and not due to a bactericidal capacity of DFP at the concentrations studied.

### Deferiprone impacts on iron content and iron metabolism of fish

**Figure 5:**
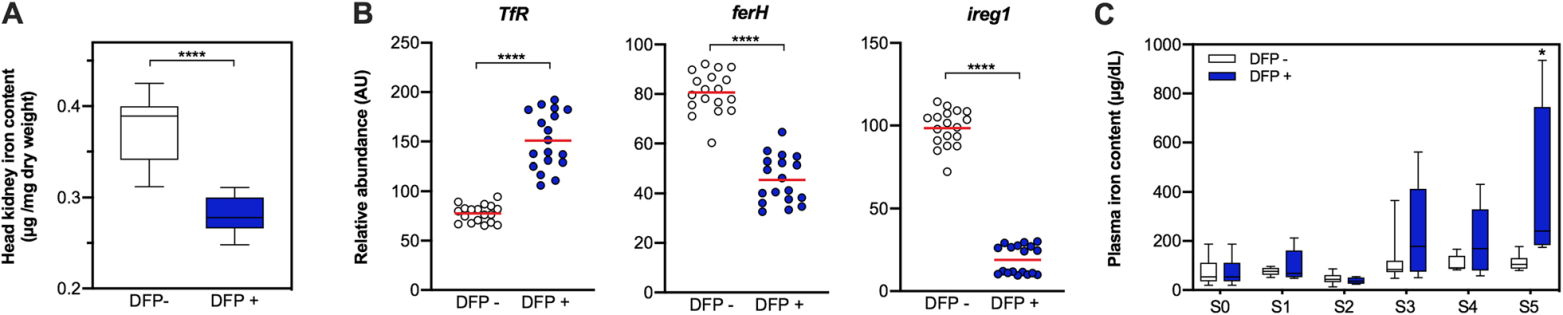
**Iron content in head kidney and plasma of** ***Oncorhynchus*** **and gene expression of iron metabolism markers after Deferiprone treatment**. **A** Determination of iron in head kidney (µg iron per mg dry weight). Iron levels in non-challenged and untreated (DFP −, white box) or DFP treated (DFP + , blue box) head kidneys were measure at S5 sampling points. Data show box and whisker plot and solid horizontal lines indicate the median and the 25^th^ 75^th^ interquartile range level of at least four fish in two independent experiments. **B** Gene expression analysis performed in non-challenged and untreated (DFP −, white circles) or DFP treated (DFP + , blue circles) fish at S5 sampling point (30 days of treatment). Transferrin receptor (*TfR*), ferritin (*ferH*) and iron-regulated transporter 1 (*ireg1)* genes were quantified relative to elongation factor 1-alpha a housekeeping gene (AU, arbitrary units). Data show mean ± SD of six fish in triplicates measured in at least two independent experiments. **C** Quantification of plasmatic iron levels in non-challenged and untreated (DFP −, white box) or DFP treated (DFP + , blue box) fish during the course of the experiment at different S0 to S5 sampling points. Data show box and whisker plot and solid horizontal lines indicate the median and the 25th 75th interquartile range level of at least four fish in two independent experiments. For **A–C**, unpaired t-test was performed; asterisks show statistically significant differences between DFP − and DFP + group denote: *p-value < 0.05, ****p-value < 0.0001.

To evaluate the effect of DFP on the iron content of fish, we quantified the concentration of iron in head kidney of unchallenged fish treated or not with DFP. As shown in Figure 5A, head kidney iron content in the DFP treated group significantly decreased compared with the untreated group (p-value < 0.05), suggesting a direct effect of the iron chelator over the abundance of head kidney iron content in fish at the end of the trial. Complementarily, we measured and compared the relative abundance of three markers of cellular iron status: transferrin receptor (*TfR*), ferritin (*ferH*) and iron-regulated transporter ferroportin 1 (*ireg1*) in head kidneys of DFP treated and untreated fish at S5 sampling point. Interestingly, as shown in Figure 5B, *TfR* showed a significant increased abundance when compared to the untreated fish, while *ferH* and *ireg1* presented a significant decreased abundance in response to the iron chelator relative to the control. Finally, we quantified the concentration of iron in the plasma of unchallenged fish treated or not with DFP. As shown in Figure 5C, plasma iron in the DFP treated group tended to increase compared with the untreated group, displaying a significant increase at point S5 (p-value < 0.05), suggesting an incremented diet absorption of iron in response to the metal chelation by DFP. These results indicate that diets supplemented with DFP at non-antibiotic concentrations, reduce the availability of iron for *P. salmonis*, which correlates with decreased bacterial proliferation and lower mortality due to SRS.

## Discussion

SRS is the most important infectious disease affecting the Chilean salmon farming industry. The high frequency of new epizootic events confirms the need to develop alternative strategies to combat this disease. The use of host-directed antimicrobial drugs (HDAD) against intracellular pathogens can be an option to avoid the use of antibiotics, and their subsequent effects on microbial resistance [9, 10, 35]. This strategy allows the perturbation of host pathways used by intracellular pathogens to proliferate inside host cells, thus avoiding infection or controlling its effects. Previous studies on the transcriptomic characterization of Atlantic salmon infected with *P. salmonis* allowed the identification of key biological processes and pathways, which are good candidates to be manipulated with these type of drugs [15, 36–39], e.g. iron metabolism [15, 38, 40]. Moreover, we reported that resistant families of Atlantic salmon are more efficient in generating iron-deprivation in infected tissues than susceptible families in response to the infection [15], emphasizing the relevance of host regulation of intracellular iron concentrations, which affects *P. salmonis* proliferation. Hence, we hypothesize that the pharmacological deprivation of cellular iron in the host may affect the intracellular proliferation of *P. salmonis* at non-antibiotic concentrations.

Currently, the U.S. Food and Drug Administration (FDA)-approved iron chelators are Deferoxamine (DFO), Deferasirox (DFX) and Deferiprone (DFP). All three available chelators are successful as monotherapy in cell systems and at clinically relevant concentrations. However, considering a practical application for the salmon farming industry, we selected DFP due to its oral bioavailability and best cost/effectiveness when compared with DFO and DFX [22], but more importantly, for its low molecular weight, neutral charge and lack of extreme hydrophilicity [25]. These features allow the drug to enter the cells, access the labile iron pool and exit rapidly as the DFP-iron complex, being the best iron chelator for shuttling purposes [41, 42].

Remarkably, our results indicate that DFP decreases intracellular iron content in macrophages-like (in vitro) and head kidney (in vivo) of salmonids at non-bactericidal nor cytotoxic concentrations (Figures 1, 4, 5). As expected, this iron reduction correlated with a decrease in intracellular bacterial proliferation and an increase in the protective capacity against infection with *P. salmonis* in cell cultures, a result that we also were able to recapitulate at a full organism level in rainbow trout (Figures 2, 3). This finding emphasizes the contribution of cellular models as an approach to evaluate the effects of drugs, such as DFP in future applications in whole organisms. Specifically, ten days post-infection, SHK-1 cells treated with DFP showed a reduced cytopathic effect of vacuolization (infection progression), bacterial load and mortality with respect to infected cells non-treated with DFP (Figure 1). These results led us to formulate diets supplemented with DFP and to evaluate its effect in vivo, by assessing two concentrations and two delivery strategies (Additional file 2). Interestingly, although all the diets supplemented with DFP showed a degree of protection against infection with *P. salmonis*, the best results were at the lowest dose and longest administration time with the iron chelator (Figure 3). These antecedents highlight that the pharmacological modulation of the host iron levels must be done with fine-tuning, considering the risk of iron depletion for fundamental processes and traits such as growth (Additional files 7 and 8).

Furthermore, it is noteworthy that a reduction in dietary iron availability would not allow obtaining comparable results to those reported with the addition of DFP, since fish deploy their iron capture systems in response to a deficit by gills and intestinal iron absorption [43]. A proof of this physiological response is given in the results shown in Figure 5: DFP-induced iron deficiency is sensed at the cellular level, where a correlation for an increase in iron cellular uptake receptor (TfR) and a decreased level of storage and efflux of cellular iron receptors (ferH and ireg1) was observed in the head kidneys of trout. Moreover, the incremented iron level in plasma suggests a systemic response to iron deficiency, which could be explained by increased intestinal absorption of the metal. Despite this physiological response, the constant input of dietary DFP allows continually reducing the intracellular iron availability to *P. salmonis*.

Given the relevance of iron for the infection process, the use of iron chelators as therapeutic agents against pathogens is not an original idea [44–49]. It has been reported that DFP can inhibit the growth of bacterial human pathogens such as *Yersinia enterocolitica* [50], *Vibrio vulnificus* [51] and coagulase-negative staphylococci [46] by decreasing iron-availability in vitro. Also, DFP is capable of inhibiting the replication of human immunodeficiency virus type 1 (HIV-1) in mononuclear blood cells [23], and treatment with higher doses of DFP has been shown to extend survival after HIV-1 infection [52]. Similar to our results, but in a mouse model of mucormycosis, it was also observed that a dietary dose of 100 mg/Kg per day of DFP improves survival when compared with non-treated animals [53]. Moreover, one trial involving 45 patients with malaria treated with DFP, showed significantly faster coma recovery and parasite clearance together with no adverse effects [54], demonstrating the versatility of this chelator as a host-directed antimicrobial drug.

On the contrary, previous studies have indicated that humans and mice treated with DFO show higher susceptibility to vibriosis, yersiniosis, salmonellosis and mucormycosis than those not treated with the iron chelator [25, 53, 55, 56]; thus, DFO may act as a siderophore for some microbes of clinical relevance, exacerbating infections. These differences between DFP and DFO could be explained by their origin, since DFP is a synthetic iron chelator while DFO is present in the nature and is currently produced from *Streptomyces pilosus* [17, 57]. Furthermore, preliminary clinical toxicity evidenced in humans suggests that DFO and DFX can only be used for non-iron overloaded conditions for short-term treatments (weeks ranges); whereas DFP can be used for longer-term treatments spanning several months [25]. These results emphasize the relevance of selecting an adequate iron chelator as a therapeutic agent, focusing its choice on physicochemical and pharmacological aspects, and also on biological characteristics of both the pathogen and host.

To the best of our knowledge, this is the first report of the protective capacity of an iron chelator against infection in fish. However, a recent study reported the effects of an induced-iron overload in Atlantic salmon during *P. salmonis* infection and the use of DFO mesylate salt as a tool to reduce the iatrogenic iron overload [58]. The results indicate that although the challenged fish treated with DFO mesylate salt did not show an improvement in survival with respect to the control, the bacterial load was significantly reduced in these fish, highlighting the critical role of iron for *P. salmonis* replication during the infection. Although our results demonstrate that DFP was able to control the replication of *P. salmonis* at non-antibiotic concentrations (Figures 2, 3), we cannot discard that DFP directly exerts an inhibitory activity on the bacterial virulence, or indirectly stimulates the immune response and/or the antioxidant capacity of the fish by systemic reduction of iron excess [59, 60]. Finally, given the increasing prevalence of antibiotic resistance, our results collectively offer the possibility to combat SRS and other fish infections in an efficient and sustainable form by using dietary DFP as a monotherapy or as an adjuvant therapy with lower doses than currently used antibiotics.

## Supplementary information

**Additional file 1.** Ingredient formulation and nutrient composition of experimental diets.

**Additional file 2.** Experimental strategy for Deferiprone delivery and sampling.

**Additional file 3.** Lethal Dose 50 determination in *Oncorhynchus mykiss* after intraperitoneal inoculation of *Piscirickettsia salmonis*.

**Additional file 4.** List of primers used in this paper.

**Additional file 5.** Efficacy indicators in fish for the different Deferiprone treatments.

**Additional file 6.** Efficacy indicators in fish for the different Deferiprone treatments.

**Additional file 7.** Effect of Deferiprone supplementation on fish percentage of ration consumption and weight gain.

**Additional file 8.** Effect of Deferiprone supplementation on survival of *Oncorhynchus mykiss*.

## Data Availability

The datasets during and/or analyzed during the current study are available from the corresponding author on reasonable request.

## Abbreviations

AC: after challenge
ANOVA: analysis of variance
ARR: absolute risk reduction
ATCC: American Type Culture Collection
BC: before challenge
CPE: cytopathic effect
DFO: deferoxamine
DFP: deferiprone
DFX: deferasirox
dpc: days post-challenge
ECACC: European Collection of Authenticated Cell Cultures
EF1A: elongation factor 1 alpha gene
ferH: ferritin
HDAD: host-directed antimicrobial drugs
HDT: host-directed therapy
HIV-1: human immunodeficiency virus type 1
IP: intraperitoneal
ireg1: iron-regulated transporter ferroportin 1
k: carrying capacity
LD_50_: median lethal dose
MOI: multiplicity of infection
NNT: number of animals necessary to treat
OD: optical density
PDAD: pathogen-directed antimicrobial drug
PPT: parts per thousand
RPS: relative percent survival
S: sampling
SRS: salmonid rickettsial septicaemia
TCID_50_: tissue culture infective dose 50
TfR: transferrin receptor
