# Supplementary material for: Pharmacological iron-chelation as an assisted nutritional immunity strategy against Piscirickettsia salmonis infection
Source: Vet Res. 2020 Oct 28;51:134. doi: 10.1186/s13567-020-00845-2 (PMC7592559; doi:10.1186/s13567-020-00845-2)
Supplement: Supplementary file 1 — Additional file 1. Ingredient formulation and nutrient composition of experimental diets. [file 13567_2020_845_MOESM1_ESM.docx]

**Additional file 1. Ingredient formulation and nutrient composition of experimental diets.**

|  | | | DFP - | DFP 50 | | DFP 100 |
| --- | --- | --- | --- | --- | --- | --- |
| Ingredient Formulation (wet basis, grams) | | | | | |  |
|  | | | | | |  |
| Fish meal | | 677 | | | 677 | 677 |
| Astaxanthin | | 0.5 | | | 0.5 | 0.5 |
| Smolt premix* | | 75 | | | 75 | 75 |
| Salt (NaCl) | | 20 | | | 20 | 20 |
| Wheat gluten | | 20 | | | 20 | 20 |
| Wheat flour | | 80 | | | 80 | 80 |
| Fish oil | | 127.5 | | | 127.5 | 127.5 |
| Sum | | 1000 | | | 1000 | 1000 |
| Deferiprone** |  | 0 | | | 5 | 10 |
|  |  |  | | |  |  |
| Nutrient Composition (dry basis) | | | | |  |  |
| Moisture (%) | | 7.1 ± 0.2 | | | 7.0 ± 0.4 | 7.1 ± 0.3 |
| Protein (%) | | 48.6 ± 0.3 | | | 48.4 ± 0.5 | 48.7 ± 0.5 |
| Fat (%) | | 22.2 ± 1.5 | | | 21.8 ± 0.5 | 21.9 ± 0.7 |
| Ash (%) | | 10.7 ± 0.1 | | | 10.4 ± 0.2 | 10.5 ± 0.4 |
| Gross Energy (MJ/kg) | | 23.4 ± 0.3 | | | 23.2 ± 0.5 | 23.3 ± 02 |
|  | |  | | |  |  |

*Supplies the following per kg dry diet: KI: 1.9 mg; MnSO_4_^.^H_2_O: 75.8 mg; ZnSo_4_^.^7H_2_O: 132.0 mg; Na_2_SeO_3_: 0.88 mg; CoCl_3_^.^6 H_2_O: 4.0 mg; CuSO_4_^.^H_2_O: 11.8 mg; FeSO_4_^.^H_2_O: 298.5 mg. Thiamin mononitrate: 62 mg; riboflavin: 71 mg; niacin: 294 mg; calcium pantothenate: 153 mg; pyridoxine hydrochloride: 50 mg; folic acid: 22 mg; vitamin B_12_: 0.08 mg; d-biotin: 0.8 mg; myoinositol: 176 mg; retinal acetate: 8818 IU; vitamin D_3_: 588 mg; α-tocopherol acetate: 670 mg; menadione sodium bisulfite complex: 37 mg.** The concentration of Deferiprone corresponds to the amount (g) incorporated in the preparation of the diets.
