# Supplementary figures and images for: Pharmacological iron-chelation as an assisted nutritional immunity strategy against Piscirickettsia salmonis infection
Source: Vet Res. 2020 Oct 28;51:134. doi: 10.1186/s13567-020-00845-2 (PMC7592559; doi:10.1186/s13567-020-00845-2)

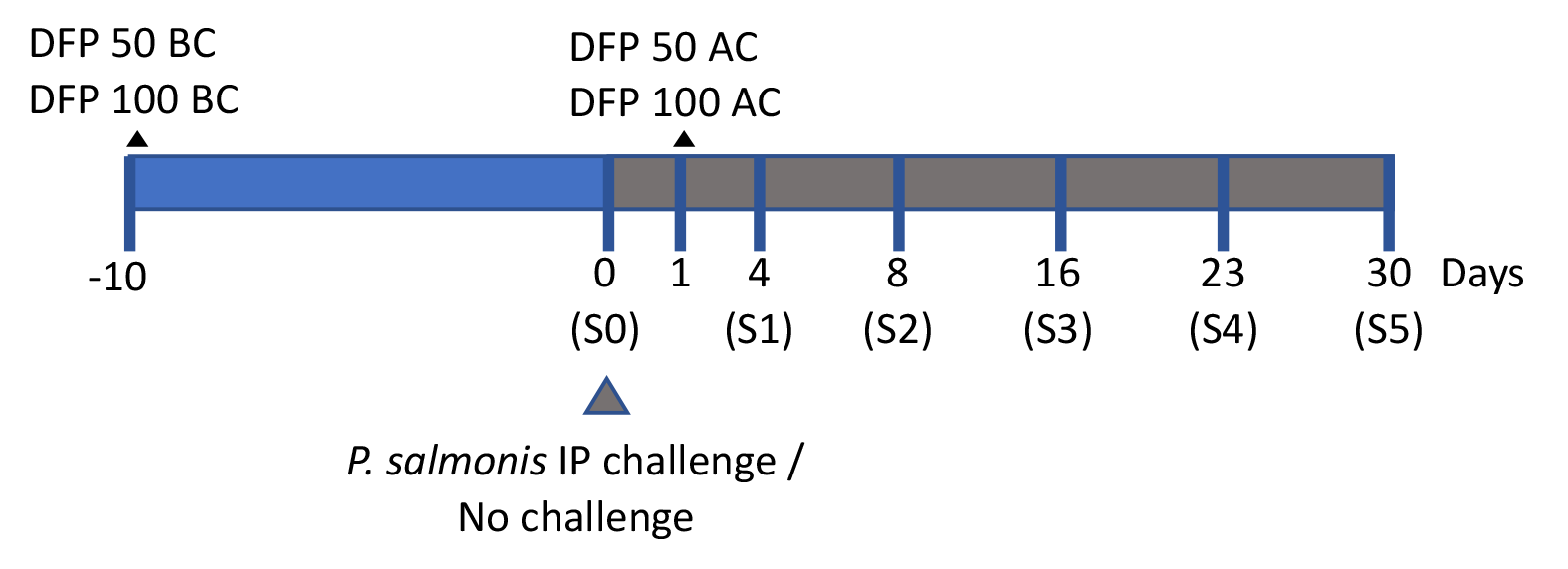

Supplement: Supplementary file 2 — Additional file 2. Experimental strategy for Deferiprone delivery and sampling. [file 13567_2020_845_MOESM2_ESM.tif]

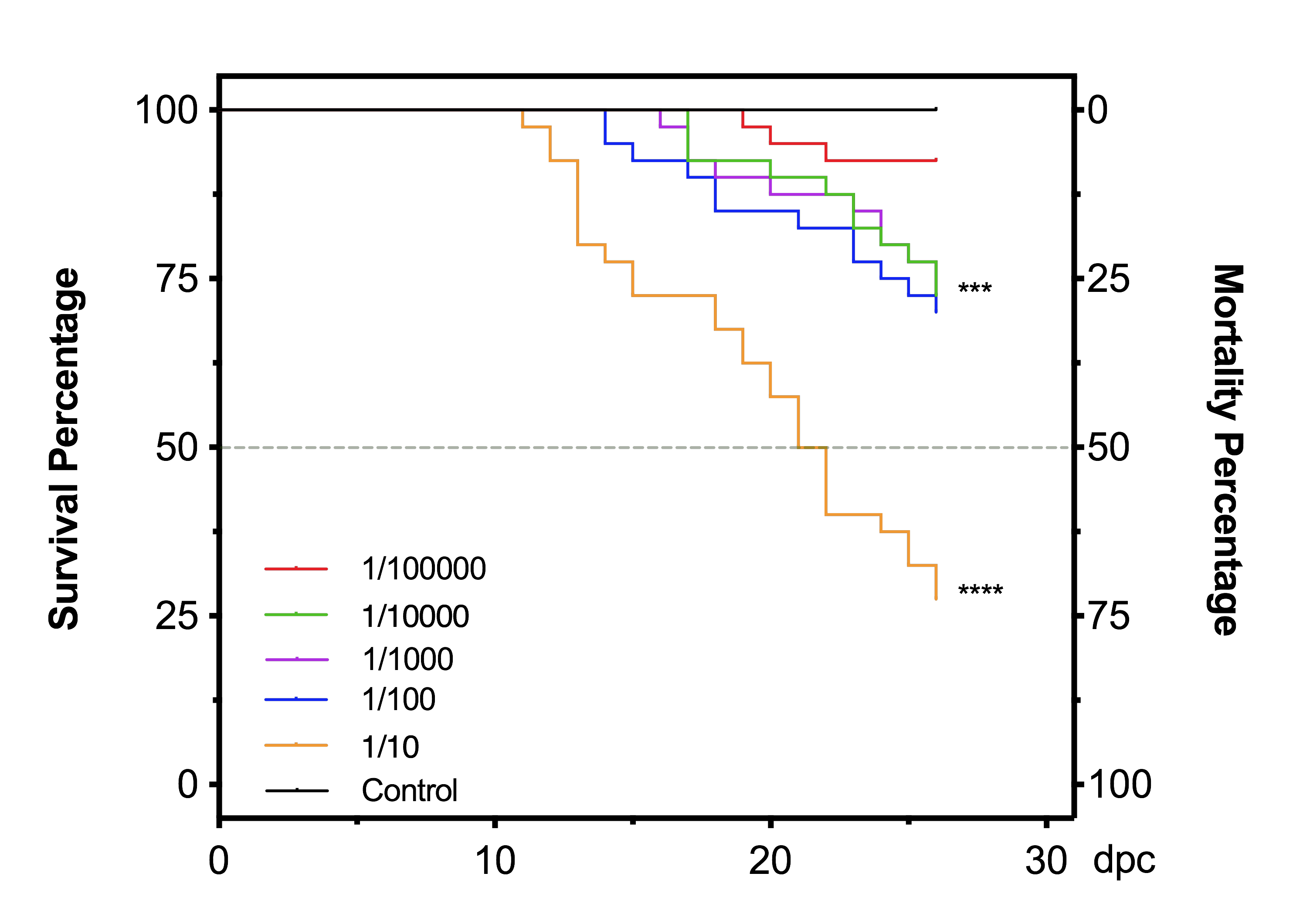

Supplement: Supplementary file 3 — Additional file 3. Lethal Dose 50 determination in Oncorhynchus mykiss after intraperitoneal inoculation of Piscirickettsia salmonis. [file 13567_2020_845_MOESM3_ESM.tiff]

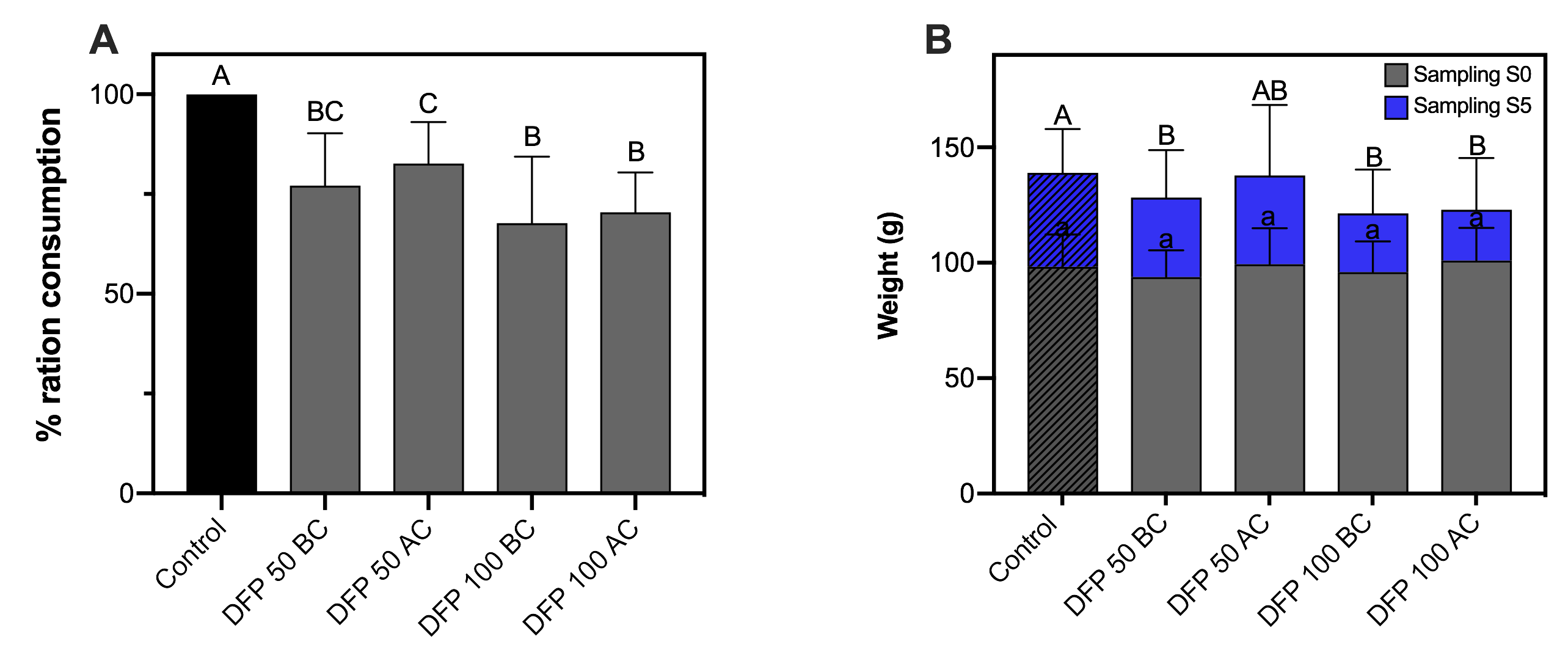

Supplement: Supplementary file 7 — Additional file 7. Effect of Deferiprone supplementation on fish percentage of ration consumption and weight gain. [file 13567_2020_845_MOESM7_ESM.tiff]

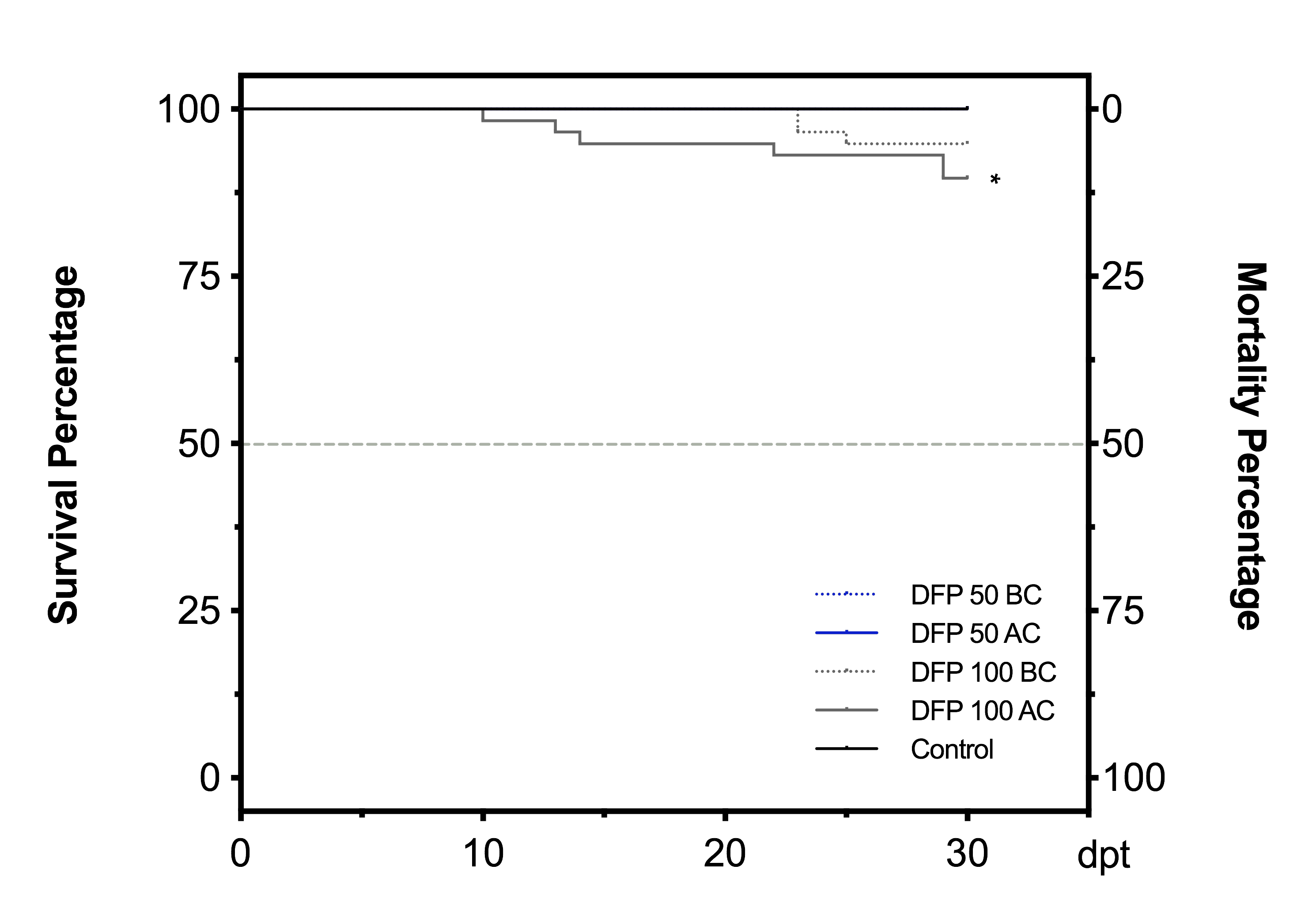

Supplement: Supplementary file 8 — Additional file 8. Effect of Deferiprone supplementation on survival of Oncorhynchus mykiss. [file 13567_2020_845_MOESM8_ESM.tiff]
