# Supplementary material for: Pharmacological iron-chelation as an assisted nutritional immunity strategy against Piscirickettsia salmonis infection
Source: Vet Res. 2020 Oct 28;51:134. doi: 10.1186/s13567-020-00845-2 (PMC7592559; doi:10.1186/s13567-020-00845-2)
Supplement: Supplementary file 4 — Additional file 4. List of primers used in this paper. [file 13567_2020_845_MOESM4_ESM.docx]

**Additional file 4.** List of primers used in this paper

| Primers | Species | Sequence forward 5´-3´ | Sequence reverse 5´-3´ |
| --- | --- | --- | --- |
| *16S rRNA* | *Piscirickettsia salmonis* | AGGGAGACTGCCGGTGATA | ACTACG AGGCGCTTTCTCA |
| *EF1α*  *TfR*  *FerH*  *Ireg1* | *Oncorhynchus mykiss*  *Oncorhynchus mykiss*  *Oncorhynchus mykiss*  *Oncorhynchus mykiss* | CACCACCGGCAATCTGATCTACAA  ACCGTTTGTCTCCCCCAAAG  CGTCAAGAAACCAGAGAAGGA  GTCCTCTTACTGGGCGCTAT | TCAGCAGCCTCCTTCTCGAACTTC  TTCCTGGTCACCGTTATGACA  AGGTAGTGTGTCTCAATGAAGTC  GCCAGGTTAGCGATGTTAGC |
