# Supplementary material for: Pharmacological iron-chelation as an assisted nutritional immunity strategy against Piscirickettsia salmonis infection
Source: Vet Res. 2020 Oct 28;51:134. doi: 10.1186/s13567-020-00845-2 (PMC7592559; doi:10.1186/s13567-020-00845-2)
Supplement: Supplementary file 5 — Additional file 5. Efficacy indicators in fish for the different Deferiprone treatments. [file 13567_2020_845_MOESM5_ESM.docx]

**Additional file 5.** **Mathematical formulas used to evaluate effectiveness parameters of the different treatments**

| **Test name** | **Formula** |
| --- | --- |
| Relative Percent Survival (RPS) | $1- \frac{Cumulative mortality of treated group}{Cumulative mortality of control group non treated} x 100$ |
| Absolut Risk Reduction (ARR) | $\left( \left( \frac{Cummulative mortality of treated group}{Total of treated group} \right)-\left( \frac{Cummulative mortality of non treated group}{Total of non treated group} \right) \right)x 100$ |
| Number of animals necessary to treat (NNT) | $\left( \frac{1}{ARR} \right)*100$ |
