# Supplementary material for: Pharmacological iron-chelation as an assisted nutritional immunity strategy against Piscirickettsia salmonis infection
Source: Vet Res. 2020 Oct 28;51:134. doi: 10.1186/s13567-020-00845-2 (PMC7592559; doi:10.1186/s13567-020-00845-2)
Supplement: Supplementary file 6 — Additional file 6. Efficacy indicators in fish for the different Deferiprone treatments. [file 13567_2020_845_MOESM6_ESM.docx]

**Additional file 6. Efficacy indicators in fish for the different deferiprone treatments**

| **Indicators** | **DFP 50 BC** | **DFP 50 AC** | **DFP 100 BC** | **DFP 100 AC** | **Control** |
| --- | --- | --- | --- | --- | --- |
| **% Mortality** | 59.6 | 81 | 68.9 | 85.7 | 91.6 |
| **% RPS** | 34.93 | 11.57 | 24.78 | 6.44 |  |
| **% ARR** | 32.63 | 11.36 | 23.46 | 6.59 |  |
| **CI 95% ARR** | 18.81 to 46.45 | -0.31 to 23.02 | 10.15 to 36.76 | -4.21 to 17.39 |  |
| **NNT** | 4 | 9 | 5 | 16 |  |
| **CI 95% NNT** | 2.2 to 5.3 |  | 2.7 to 9.9 |  |  |

Indicators calculated in relation to control group.

RPS: Relative percentage survival, ARR: Absolute risk reduction, NNT: Number necessary to treat, CI: Confidence interval.
